# Statistics of the Medical Profession in Norway

**Published:** 1843-01

**Authors:** 


### Statistics of the Medical Profession in Norway.

In 1840 there were in Norway 128 civil and 58 military medical practitioners, of whom only 9 (military ones) had undergone no medical examination. In 1816 the number of practitioners was only 99; in 1824, 116; in 1833, 129; in 1837, 148; and in 1839, 159. The numbers of those who had been examined were in 1816, 71; in 1824, 86; in 1833, 95; in 1837, 128; in 1839, 149; and in 1840, 177. The number of practitioners has thus increased 88 per cent. in 24 years; and the number of those after examination, 149 per cent. Since the population of the kingdom in 1816 was 900,000, and in 1840, 1,250,000, it follows that in 1816 there was one physician to every 9000 persons, but in 1840 one to every 6800.

*Norsk Magazin for Lægevidenskaben, Mars*, 1841.

Compare a report "On the state of Medicine in Norway," in vol. IV, p. 541.

